# Advanced Eye Movement Features Measured by Quantitative Oculography as Candidate Biomarkers for Progressive Supranuclear Palsy

**DOI:** 10.3390/diagnostics16182944

**Published:** 2026-09-11

**Authors:** Haoxuan Ouyang, Bo Liu, Qiwei Peng, Zhuoran Ma, Zhicheng Tang, An Chang, Maoyu Liu, Xuebing Cao, Yan Xu, Yun Xia

**Affiliations:** 1 Department of Neurology, Union Hospital, Tongji Medical College, Huazhong University of Science and Technology, Wuhan 430022, China; m202476253@hust.edu.cn (H.O.); alexpeng@hust.edu.cn (Q.P.); zhuoransigexi@163.com (Z.M.); 15978054162@163.com (Z.T.); changan1006@126.com (A.C.); lmaoyu2002@163.com (M.L.); caoxuebing@126.com (X.C.); 2 Department of Otorhinolaryngology-Head Neck Surgery, Union Hospital, Tongji Medical College, Huazhong University of Science and Technology, Wuhan 430022, China; liuboent@hust.edu.cn

**Keywords:** progressive supranuclear palsy, advanced eye movements, saccades, oculography, diagnostic markers

## Abstract

Conventional qualitative eye movement examinations may miss subtle abnormalities, complicating early and accurate diagnosis of progressive supranuclear palsy (PSP). Quantitative oculography, represented by video-oculography (VOG), provides an objective digital assessment of eye movements. This review summarizes abnormalities in advanced eye movement tasks in PSP, including prosaccades (ProSs), antisaccades (ASs), memory-guided saccades (MGSs), predictive saccades (PSs), overlap saccades (OSs) and gap saccades (GSs), and considers their underlying pathophysiological mechanisms. Quantitative metrics from these tasks may help distinguish PSP subtypes and PSP from other neurodegenerative diseases, such as Parkinson’s disease (PD), multiple system atrophy (MSA), corticobasal degeneration (CBD) and Alzheimer’s disease (AD). Advanced eye movement measures are therefore promising candidate biomarkers for PSP diagnosis and longitudinal disease monitoring.

## 1. Introduction

Progressive supranuclear palsy (PSP) is a common atypical Parkinsonian syndrome (APS) [1]. Its five-year mortality rate reaches 30.5%, ranking first among various degenerative Parkinsonian syndromes [2]. Core clinical features include postural instability, vertical supranuclear gaze palsy, axial rigidity and bradykinesia [3,4]. PSP includes the classic PSP-Richardson syndrome (PSP-RS) and several clinical variants, which comprise PSP-predominant Parkinsonism (PSP-P), PSP-progressive gait freezing (PSP-PGF), PSP-predominant cerebellar ataxia (PSP-C), PSP-corticobasal syndrome (PSP-CBS), PSP-frontal (PSP-F), and others [5,6]. However, in the early stages, most PSP patients present only with lower limb weakness and non-specific bradykinesia, which is easily confused with idiopathic Parkinson’s disease (PD). Generally, a definitive clinical diagnosis is often delayed until 3 to 4 years after the onset of the initial symptoms, when typical core signs such as recurrent falls, supranuclear gaze palsy, and the “hummingbird sign” caused by midbrain atrophy fully manifest [7]. Therefore, developing objective, quantifiable, and sensitive biomarkers applicable to the subclinical stage is a critical issue that urgently needs to be resolved in the field of early PSP diagnosis.

Eye movement abnormalities are not only the origin of the nomenclature of PSP but also a biological marker in its diagnostic system [8]. Currently, routine clinical evaluation relies primarily on bedside qualitative examinations, using visual observation of ocular pursuit and saccadic movements to determine the presence of vertical supranuclear gaze palsy [9]. However, such crude assessments can only identify lesions in the middle and late stages of the disease, when widespread and irreversible neuronal damage has already occurred, and they cannot detect occult eye movement impairment, showing clear limitations in sensitivity [10]. The emergence of advanced eye movement detection technology effectively addresses this shortcoming. Advanced eye movements are voluntary eye movements co-regulated by the cerebral cortex and subcortical eye movement centers, which are achieved through complex cognitive processing and can reflect the cognitive function and neural circuit integration capabilities of different brain regions [11]. Saccadic eye movements depend on the coordinated regulation of multiple brain regions, including the brainstem, basal ganglia (BG), and cerebral cortex, whereas PSP is characterized centrally by degenerative lesions in the brainstem and BG, leading to a high overlap in their pathological targets. The types of advanced eye movement involved in this study include prosaccades (ProSs), antisaccades (ASs), memory-guided saccades (MGSs), predictive saccades (PSs), gap saccades (GSs), and overlap saccades (OSs) [11,12,13,14].

Saccades are easily monitored and quantifiable, and standardized and individualized detection of them can be achieved by modulating the spatiotemporal parameters of visual signals, offering broad application potential in the cognitive assessment and differential diagnosis of neurodegenerative diseases [15,16]. With deepening research into neural circuits regulating eye movements and the clinical popularization of quantitative VOG, advanced eye movement examination has gradually transitioned from a laboratory research tool into a biomarker with high clinical application value. This technology can not only capture subtle eye movement abnormalities but also indirectly evaluate the degree of pathological damage to specific cerebral cortical and subcortical neural networks [17,18]. Data acquisition and calibration of oculography can be performed by trained technical personnel or allied-health professionals, including nurses and pharmacists, following a standardized protocol [19]; a specialist is not required to operate the device directly. Clinical interpretation, however, should be integrated with the neurological examination by a clinician experienced in oculomotor disorders, particularly because PSP-specific protocols and cut-offs are not yet uniform [20,21]. Based on this background, from a clinical neurology perspective, this paper reviews the current application status and quantitative characteristics of advanced saccades in PSP, analyzes their underlying neuropathological and physiological mechanisms, and discusses the application prospects and future research directions of this technology in subtype differentiation, differential diagnosis, and dynamic monitoring of disease progression.

This review adopted a selective narrative synthesis approach to summarize and discuss studies on advanced oculomotor function in PSP. We searched PubMed for relevant articles published up to 1 July 2026, using combinations of the following terms: progressive supranuclear palsy, PSP, eye movement, oculomotor, eye tracking, prosaccade, antisaccade, memory-guided saccade, memory saccade, predictive saccade, anticipatory saccade, gap saccade, and overlap saccade. We also reviewed relevant reviews and the reference lists of the included articles to identify additional eligible studies. Studies were excluded if they did not report quantitative eye movement findings in patients with PSP, mentioned eye movements without providing information related to the saccadic functions described above, were not directly relevant to the clinical features or pathophysiological mechanisms discussed in this review, or were technical demonstration studies without clinical relevance. In total, 53 oculography-based clinical studies were included in the clinical evidence synthesis. These studies used observational clinical designs, including cross-sectional, case–control, and cohort studies. Case reports, reviews, and meta-analyses were not included because this synthesis focused on original clinical studies reporting quantitative oculographic findings in patients with PSP. As this study was not a systematic review, we did not perform a formal study quality assessment or provide a structured evidence table. However, we qualitatively assessed the methodological limitations of the included studies.

## 2. Clinical Evaluation Methods for Advanced Eye Movements

Subtle abnormalities, including millisecond-level changes in saccadic latency, square-wave jerks (SWJs), and trajectory distortions of small-amplitude saccades, cannot be accurately captured by the naked eye, which greatly limits the accuracy of early PSP diagnosis. In the late 20th century, the application of electro-oculography (EOG) and infrared oculography enabled the quantitative acquisition of ocular kinematic parameters [22]. EOG utilizes the electrostatic-potential difference between the cornea and retina to record eye positions by capturing electric-field changes induced by eye rotation. Although it is susceptible to facial electromyographic interference, its simplicity has led to its widespread use in neurology wards [23]. Currently, VOG or video-nystagmography (VNG) serves as the core technology for clinical eye movement assessment and remains the mainstream tool for advanced eye movement research in PSP [9,24]. By illuminating the eye with an infrared light source and using high-speed industrial cameras to capture the relative displacement between the pupil center and the corneal reflection point in real time, this technology can automatically and precisely extract quantitative parameters, including peak saccadic velocity, latency, duration, accuracy, gain, and the main sequence curve [22,25]. The non-invasive nature, high sampling rate, and integration with real-time computer analysis systems of VOG devices enable precise analysis of ProS velocity, latency, and AS error rates (Table 1). Regarding data fitting and analysis, compared with traditional exponential equations, the power function method can accurately fit the trajectory characteristics of PSP microsaccades (amplitude less than 2°) and precisely identify the kinematic distortions of small-amplitude saccades, significantly improving the detection rate of subtle abnormalities [26].

## 3. Physiological Basis and Regulatory Mechanisms of Advanced Saccades

Advanced eye movements are voluntary eye movement paradigms distinct from lower-order reflexive eye movements such as the vestibulo-ocular reflex (VOR). Lower-order reflexive eye movements rely solely on the regulation of the midbrain and pons [27]. In contrast, advanced eye movements require the coordinated involvement of subcortical nuclei and higher cognitive cortices, such as the frontal eye field (FEF), supplementary eye field (SEF), posterior parietal cortex (PPC), dorsolateral prefrontal cortex (DLPFC), and anterior cingulate cortex (ACC). They integrate complex cognitive processes including attention, memory, inhibition, and temporal prediction [14,28,29], which reflect the functional status and circuit integration levels of multiple brain regions within the central nervous system [30,31]. The core physiological function of saccades is to rapidly and conjugately rotate the eyes, projecting visual targets onto the fovea centralis of the retina to achieve clear vision [32]. Characterized by high spatiotemporal resolution, standardizable regulation, and easily quantifiable analysis, saccades serve as an ideal vehicle for investigating the pathological mechanisms and clinical differential diagnosis of neurodegenerative diseases [33]. A summary of the six classic saccade paradigms in advanced saccades [11,12,31,34,35,36] is presented in Table 2.

**Figure 1:**
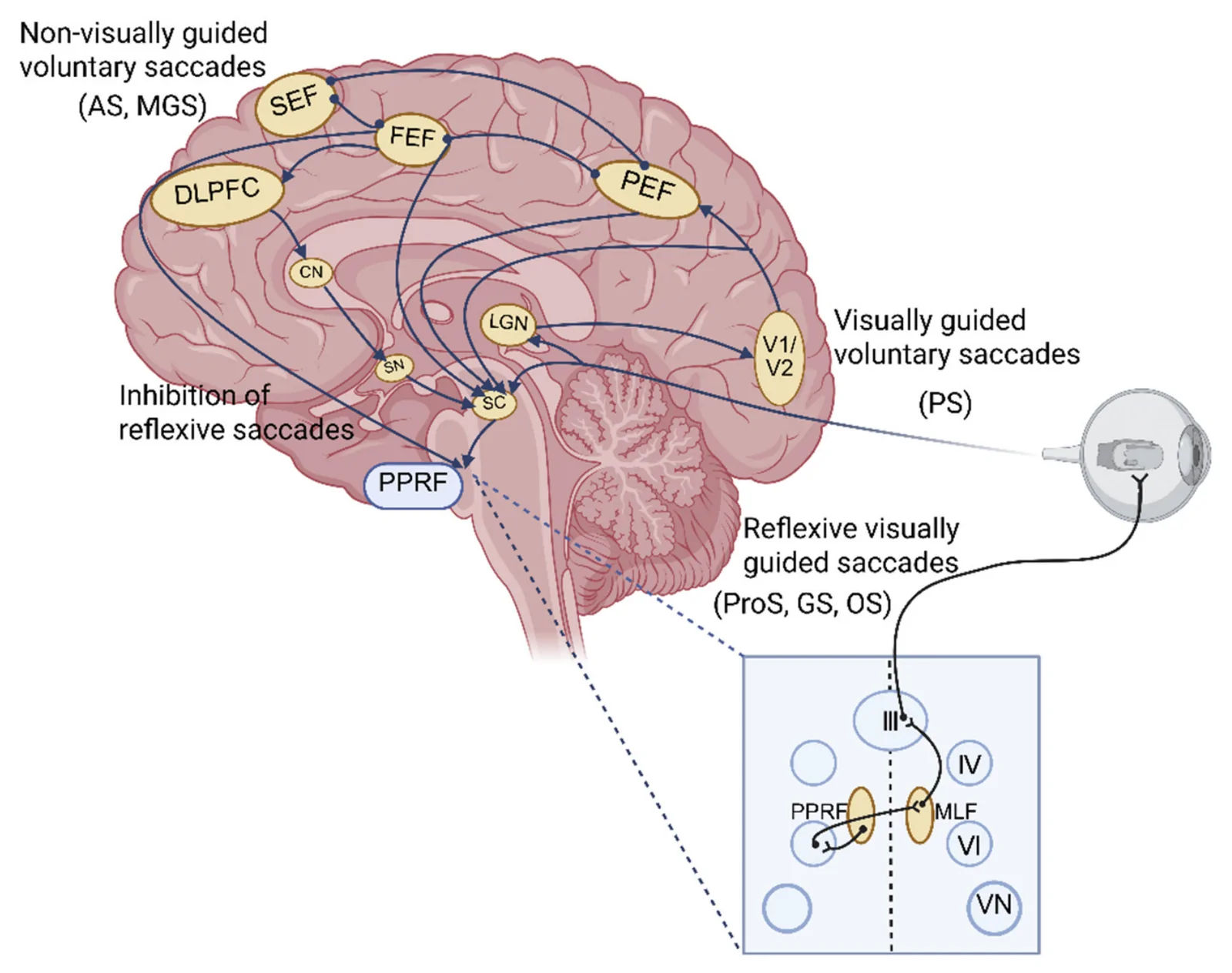
Complex cortical and subcortical neural networks regulate advanced saccades. These networks are divided into pathways for non-visually guided voluntary saccades (ASs, MGSs), visually guided voluntary saccades (PSs), and reflexive visually guided saccades (ProSs, GSs, OSs). Higher-level structures (e.g., DLPFC, FEF, SEF) exert top-down regulation and inhibition on these advanced saccades, ultimately converging signals onto the SC and the pontine PPRF to drive downstream brain nuclei to execute precise eye movements. Created in BioRender. Ouyang, H. (2026) https://BioRender.com/3uixhxl.

Precise execution of advanced saccades depends on a multi-level, sophisticated neural network comprising the cerebral cortex, BG, midbrain SC, and brainstem motor centers (Figure 1). Saccadic paradigms, governed by distinct neural circuits, fall into reflexive regulatory circuits and cognitive inhibitory regulatory circuits.

ProS is primarily mediated by reflexive visual regulation, relying chiefly on the posterior visual attentional orientation network composed of the occipital visual cortex (V1/V2), PPC, and FEF [37]. Exogenous visual target signals are transmitted through the visual cortex to the FEF, directly activating motor neurons in the intermediate and deep layers of the SC. The SC then transmits motor commands to the brainstem saccadic burst generator, rapidly converting the visual target signal into ocular muscle movement to achieve fast reflexive saccades [34,38,39]. GS and OS remain essentially reflexive, visually guided saccades, but their saccadic initiation is influenced by task structure [40].

ASs, MGSs, and PSs rely on higher cognitive regulation from the prefrontal cortex, with top-down inhibitory control and voluntary regulation. The DLPFC serves as the central control hub for executive function and impulse inhibition [41]. Through direct DLPFC-SC fibrous projections, it exerts highly precise and rapid descending inhibitory potentials on the SC, blocking reflexive saccades induced by exogenous visual stimuli and ensuring the precise execution of voluntary saccades [41,42,43]. Concurrently, the frontal-BG indirect pathway serves a gating function. Under normal conditions, the substantia nigra pars reticulata (SNpr) continuously releases GABAergic inhibitory signals to maintain fixation stability and prevent interference from irrelevant eye movements. During the execution of voluntary saccades, the FEF and DLPFC activate the caudate nucleus (CN), disinhibiting the SC via the direct pathway of the BG to trigger saccade initiation [38,39,44].

The brainstem is the final pathway for saccade execution [45]. The paramedian pontine reticular formation (PPRF) regulates horizontal saccades, whereas the rostral interstitial nucleus of the medial longitudinal fasciculus (riMLF) regulates vertical saccades [46]. During fixation periods, omnipause neurons (OPNs) in the nucleus raphe interpositus (RIP) of the pons fire at a constantly high frequency, exerting strong glycinergic tonic inhibition on the burst neurons of the riMLF and PPRF to lock the oculomotor burst generator and ensure fixation stability [47,48]. Upon saccade triggering, descending motor commands from the SC first selectively inhibit and silence OPNs by exciting long-lead burst neurons (LLBNs) [49]. This removes the inhibitory gating on the riMLF/PPRF, allowing these burst neurons to generate extremely high-frequency action potential discharges (pulse signals) [50,51], which drives the rapid contraction of extraocular muscles to achieve saccade. Additionally, the cerebellar output pathway is thought to enhance the initial pulse in the early phase of the saccade by sending signals to ipsilateral excitatory burst neurons (EBNs) and to terminate the saccade in the late phase by sending inhibitory signals to contralateral inhibitory burst neurons (IBNs) [52].

## 4. Advanced Saccadic Characteristics and Pathophysiological Mechanisms in Patients with PSP

**Figure 2:**
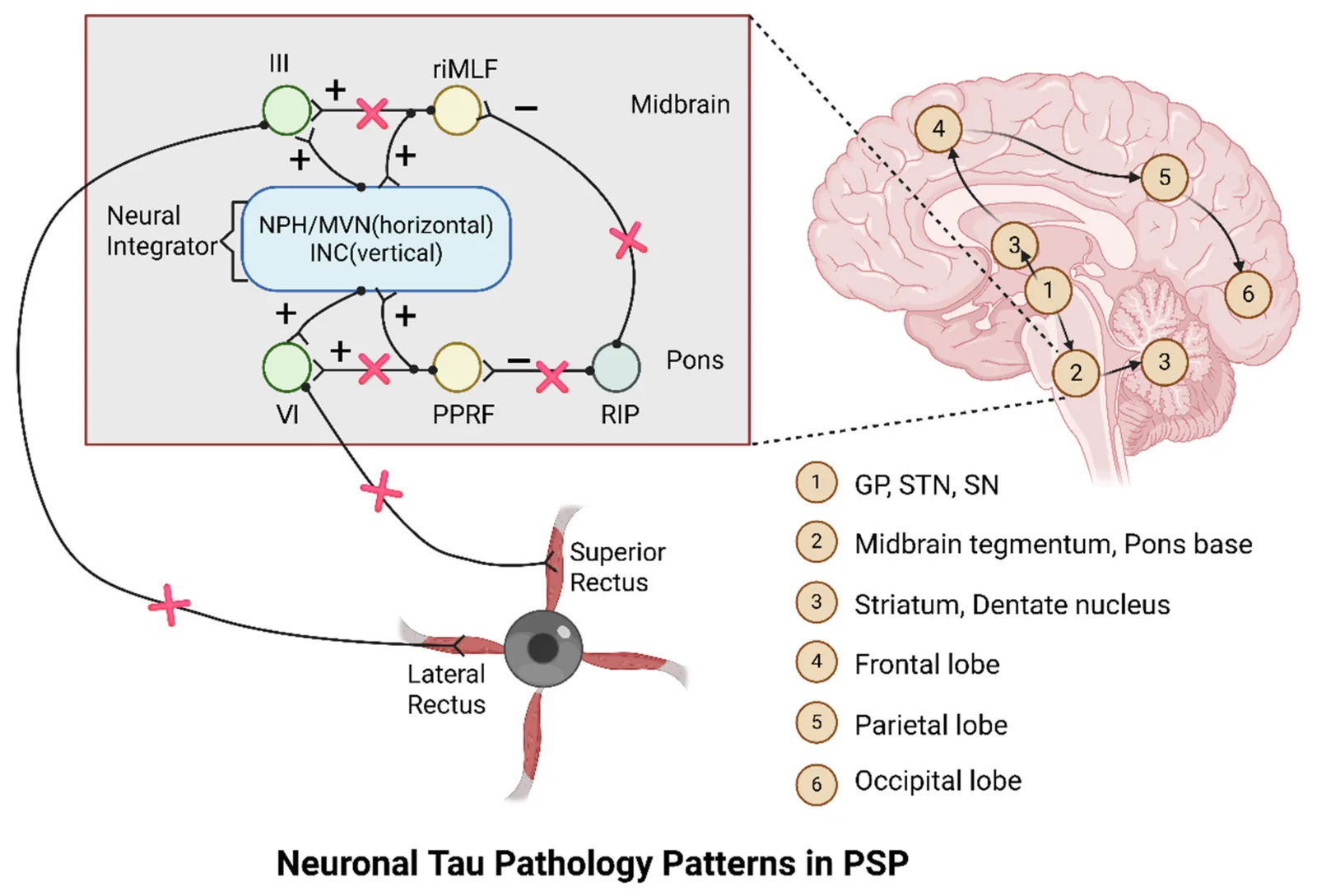
The sequential regional distribution of tau pathology in PSP has been demonstrated in postmortem neuropathological studies, with early involvement of the subcortical BG and brainstem, followed by the striatum, cerebellar dentate nucleus, and cortical regions [56,57,58]. This pathological involvement may contribute to advanced saccadic impairments. The brainstem oculomotor network is a schematic representation based largely on neurophysiological studies in primates and supported by observations in humans [59,60]. Dysfunction of the riMLF, PPRF, RIP, and neural integrators, including the INC and NPH/MVN, may extensively disrupt saccade generation, as indicated by the red crosses, and prevent the downstream oculomotor and abducens nucleus from effectively transmitting normal burst signals to the extraocular muscles. Created in BioRender. Ouyang, H. (2026) https://BioRender.com/793kw2j.

Characteristic saw-tooth (multi-step-pattern) vertical saccades accompanied by alternating horizontal components have been reported in multiple cases of PSP, along with frequent SWJs, saccadic slowing, and hypometria [53,54,55]. The pathological features of PSP include abnormal 4R-tau protein deposition as well as neuronal degeneration and necrosis in the midbrain, brainstem, BG, and prefrontal cortex (Figure 2). This involves the multi-level regulatory circuits of advanced saccades, leading to characteristic oculomotor abnormalities. Therefore, elucidating the advanced saccadic abnormalities in PSP can both reveal the pathoanatomical mechanisms of PSP and provide candidate biomarkers for its early precise diagnosis (Table 3).

### 4.1. Prosaccades (ProSs)

#### 4.1.1. Manifestations

Several previous studies evaluating horizontal saccades found that patients with early PSP exhibit small-amplitude multi-step saccades (with relatively preserved velocity for small saccades) [65,77,85]. In contrast, despite a relatively shorter latency, most saccades in late-stage PSP have very small amplitudes [65]. In healthy individuals, peak velocity rises rapidly with increasing amplitude until saturation is reached [82]. However, the capacity to increase velocity is severely impaired in PSP, leading to a significant reduction in peak velocity during large-amplitude ProS [26,92], which manifests on the saccadic trajectory as a sequence of jerks embedded in creeping movements or an initial small jerk followed by creeping movements [22]. Additionally, when PSP patients perform slow vertical ProS, the accompanying horizontal SWJs can transiently accelerate the vertical saccadic velocity, forming the clinically characteristic “round-the-houses” phenomenon [82,93]. Nevertheless, a study reported no significant abnormalities in the ProS of PSP [61].

Wunderlich et al. reported that vertical saccadic peak velocity and saccadic intrusion (SI) rate showed high specificity within their study cohort, supporting their use as level O2 and O3 diagnostic indicators for PSP in the Movement Disorder Society (MDS) di-agnostic criteria, whereas vertical saccadic velocity asymmetry was unsuitable as a sup-plementary diagnostic indicator [64]. Compared with healthy controls (HCs), PSP patients exhibit significant abnormalities in mean ProS latency [76], horizontal latency [25,55,65,80,87], horizontal peak velocity [25,52,55,66,69,72,76,77,82,90,94], horizontal amplitude [69,90,94], horizontal accuracy [65,77,87], horizontal duration [52,94] (especially the deceleration phase [52]), horizontal gain [22,55,66,76,83], vertical latency [25,55,87,89,90], vertical–horizontal latency difference [89], vertical peak velocity [22,25,55,66,69,70,72,76,82,87,90], vertical mean velocity [79], vertical amplitude [69,70,90], vertical gain [55,66,76,79], vertical accuracy [87], vertical duration [70], horizontal/vertical saccadic acceleration and deceleration [82], vertical peak velocity/mean velocity ratio [26], and SI rate [76]. However, inconsistent conclusions exist; some studies found that horizontal latency [52,73,77,83,90], horizontal peak velocity [87], directional error rate [73,90], duration [77], main sequence slope at the origin (*V*_0_/*A*_0_) [52], and the core oculomotor parameters of small-amplitude saccades [52,81] do not differ from those of HC.

Compared with PD, PSP patients show a significantly greater degree of abnormality in ProS accuracy [65,87] (especially vertical accuracy [87,91]), vertical amplitude [70,72], horizontal peak velocity [22,25,68,76,91], vertical peak velocity [22,25,26,68,70,76,87,91], mean latency [76], horizontal latency [25], vertical latency [25,89], vertical–horizontal latency difference [89], horizontal gain [22,76], vertical gain [76] (especially downward gain [22]), vertical duration [26], and SI rate [76]. In particular, the PSP-RS subtype exhibits a significantly greater prolongation of vertical ProS latency, reduction in horizontal ProS peak velocity and accuracy, and incidence of SWJs compared with PD [91]. Macro-SWJs may be observed only in a minority of patients with PSP-RS and are rare in other subtypes as well as in PD [91]. Other studies have found no significant differences between PSP and PD in horizontal saccadic hypometria rate (i.e., accuracy), horizontal peak velocity [87], horizontal saccadic mean velocity [79], horizontal saccadic latency [87,89,91], horizontal saccadic gain [79], and main sequence parameters [67,70], particularly in PSP-PGF [91]. However, prolonged horizontal/vertical latency, low-gain saccades, and SIs during ProS in PSP have also been reported to be not entirely different from those observed in PD or multiple system atrophy (MSA) [55]. This discrepancy may be explained by the fact that all patients in that study were assessed while receiving antiparkinsonian medication. Previous studies have shown that levodopa treatment significantly improves maximum oculomotor range in PD, whereas oculomotor range remains markedly restricted in PSP or MSA [95]. Although horizontal ProS accuracy may be comparable between PSP and PD in the early disease stages, ProS accuracy deteriorates significantly more rapidly in PSP than in PD as the Hoehn and Yahr (HY) stage advances [65]. One study found that the horizontal ProS maximum quick-phase velocity (MQPV) in patients with PSP was lower than in those with PD [67]. Interestingly, this finding remained valid even after excluding patients with the PSP-C.

Quattrone et al. found that PSP-RS often presents with bidirectional vertical saccadic impairment involving both upward and downward directions, whereas PSP-P mostly manifests only as upward gaze dysfunction, specifically reflected in the product of vertical saccadic amplitude × peak velocity (AxV) [17]. This is similar to the clinical observation by some physicians that upward saccadic involvement in early PSP may be more severe than downward involvement [96]. Consistent with this, Becker et al. found that vertical upward ProS peak velocity has excellent diagnostic ability for differentiating PSP, which remains unaffected by disease duration [66]. If the vertical upward ProS peak velocity is combined as a parametric product with the gain of the vertical downward 10° target ProS, the discriminative power is further enhanced [66].

#### 4.1.2. Pathophysiological Mechanisms of Abnormal Manifestations

In the early stages of PSP, EBNs in the riMLF and LLBNs responsible for feedback and trigger regulation in the midbrain tegmental area undergo highly dense tau protein deposition, vacuolar degeneration, and extensive death [47,48,51,97]. This leads to a sharp decline in the firing frequency of the vertical oculomotor center, rendering it unable to provide high-frequency burst-like action potential inputs to the oculomotor nucleus, which manifests as a precipitous and symmetrical decrease in vertical saccadic peak velocity. While structures such as the midbrain riMLF are severely damaged, the pontine PPRF region controlling horizontal saccades is relatively preserved during early pathological invasion [9,48]. In the late stages, degeneration of PPRF neurons causes a decline in horizontal peak velocity and accuracy as well.

Additionally, the midbrain reticular formation (MRF) sends commands to the riMLF and PPRF. The degeneration of the MRF in PSP can also lead to saccadic slowing [51,98]. To accomplish the intended gaze shift, saccades with reduced peak velocity in PSP must be compensated for by a lower deceleration per unit time and a prolonged deceleration duration [52]. Degeneration of the cerebellar efferent pathways in PSP can simultaneously prolong both the acceleration and deceleration phases [99].

The slow eye movements in PSP patients stem not only from reduced saccadic velocity but also from irregular saccadic trajectories and interruptions during the saccade [82]. This occurs because premature or asynchronous discharging of IBNs in PSP patients leads to premature activation of the SC fixation zone via feedback. In the RIP of PSP, OPNs exhibit neuronal loss and abundant neurofibrillary tangles, and the perineuronal nets (PNNs) are severely degraded or fragmented. This structural disruption of the gating element causes abnormal premature discharging of OPNs during saccade execution. Dysregulation of OPNs can result in delayed saccade initiation, decreased synchrony, and irregular trajectories [82,88,100]. When the vertical burst is insufficient to overcome OPN inhibition, PSP patients may utilize a horizontal burst to inhibit OPNs, thereby forming a saw-tooth trajectory or “round-the-houses” phenomenon [53]. This also explains why vertical saccades in PSP can be transiently accelerated when accompanied by SWJs.

### 4.2. Overlap Saccades (OSs)

#### 4.2.1. Manifestations

Under overlap conditions with intense visual conflict, PSP patients exhibit a significant prolongation of vertical OS latency [75] and a decrease in horizontal OS accuracy [85]. Furthermore, the mean velocity, first gain, and final gain (excluding corrective saccades) of both horizontal and vertical OS are significantly reduced in PSP, and the reductions in mean velocity and first gain are far more severe than those observed in corticobasal syndrome (CBS) and Alzheimer’s disease (AD) [75]. However, some studies suggest that the peak velocity of PSP during horizontal OS tasks does not differ from that of age-matched healthy individuals [85]. Fisk et al. also found that the initiation capability of a single horizontal OS in PSP may be largely normal regardless of large or small amplitudes. Nevertheless, under the stress of overlapping stimuli, PSP patients show a higher proportion of hypometria than HC when facing horizontal targets with large eccentricities [81].

#### 4.2.2. Pathophysiological Mechanisms of Abnormal Manifestations

Due to the deposition of tau proteins in the FEF, PEF, and BG-SC pathways of PSP patients, the rostral fixation zone of the SC and the pontine OPNs remain in a continuous state of high-frequency discharge, making disengagement from fixation extremely difficult. In the OS paradigm, because the central fixation point remains illuminated, the PPC must actively generate a visual attention disengagement signal to shift attention to the periphery. In PSP, owing to widespread cerebral cortical atrophy and reduced metabolic rates in the posterior parietal cortex, the active attention disengagement pathway is blocked, leading to prolonged OS latency [101,102].

### 4.3. Gap Saccades (GSs)

#### 4.3.1. Manifestations

Under 10° horizontal and 25° horizontal gap conditions, the GS latency in PSP can present as normal [75,84] or prolonged [103]. In contrast, with a horizontal 20° target, the GS peak velocity and accuracy are reduced in PSP [85]. Patients with PSP exhibit significant decreases in horizontal GS peak velocity, accuracy, and asymmetry early in the disease course, and some patients also present with SWJs [84,86]. Because the gap effect eliminates part of the active fixation pressure, the latency of the GS task in PSP may be shorter than that of the OS task [86].

#### 4.3.2. Pathophysiological Mechanisms of Abnormal Manifestations

The disappearance of the central fixation point removes the active fixation pressure and allows for the passive attenuation and release of OPNs, which may preserve the GS latency at small eccentricities. The gap condition in the GS paradigm induces spontaneous deactivation of OPNs, and the fixation zone in the superficial layers of the SC also undergoes spontaneous resting discharge (passive disengagement). This lowers the threshold for triggering saccades, thereby reducing high dependence on the active attention disengagement network of the PPC [102]. PSP patients may still utilize this relatively preserved subcortical disengagement mechanism during the early stages, until global degeneration of the SC occurs in the late stage of the disease. However, this does not imply that the entire advanced saccadic system of PSP remains intact. Like OS, the reductions in GS peak velocity and accuracy remain primarily driven by insufficient burst signals resulting from the degeneration of the brainstem saccadic burst generator (riMLF/PPRF) and the MRF, accompanied by early SWJs due to impaired vestibulocerebellar modulation of oculomotor feedback.

### 4.4. Antisaccades (ASs)

#### 4.4.1. Manifestations

In a prospective registry study, Painous et al. found that in the suggestive PSP (so-PSP) group, worse AS metrics at baseline combined with a negative or low-fluorescence positive alpha-synuclein seed amplification assay (asyn-SAA) were associated with increased diagnostic certainty during the follow-up period [61]. Since the definition of so-PSP excludes macroscopically visible clinical oculomotor abnormalities, video-electro-oculography (VEOG) may become a potentially important tool for screening the so-PSP population and achieving ultra-early diagnosis in future clinical trials.

The percentage of spontaneously corrected AS errors is significantly reduced in PSP [75], and the horizontal AS error rate [73,83,84,103] and AS correct response latency are significantly increased [73,103]; these features are considered key characteristics for the diagnosis of PSP. Additionally, in delayed AS tasks, the proportion of correct responses is reduced, the proportion of anticipatory prosaccades during delayed AS is increased, and the correct response latency of delayed AS is prolonged in PSP [103]. However, some studies have noted that the horizontal and vertical AS latency [75,90], horizontal AS amplitude [90], vertical AS peak velocity [90], and directional error rates of horizontal and vertical AS [90] in PSP do not differ from HC. This discrepancy may be due to inconsistent definitions of the saccadic onset across different studies, or because the AS tasks were presented in an alternating manner, which presents a certain level of difficulty even for healthy individuals.

Notably, in a mixed-task study using randomly alternating horizontal AS and GS instructions, the error rate of PSP under the mixed-task condition did not differ significantly from that during single-task performance [73]. Furthermore, the mixing cost of error rates (calculated as the saccadic error rate of repeat trials in the mixed task minus the single-task ProS error rate) was significantly elevated in PD and corticobasal degeneration (CBD), whereas the mixing cost in PSP was normal [73].

#### 4.4.2. Pathophysiological Mechanisms of Abnormal Manifestations

When performing highly difficult multi-task switching, PSP patients do not experience an additional cognitive load from the switching itself; their AS error rate stems primarily from the impairment of baseline inhibitory mechanisms [73]. Widespread prefrontal hypoactivation leads to severe impairment of the active inhibition exerted by the DLPFC on the caudal SC [104]. When a peripheral target is illuminated, exogenous visual stimuli easily bypass this defective inhibitory barrier and are transmitted directly through the parietal PEF to the SC, thereby triggering an uncontrollable visual grasp reflex and manifesting as persistently high AS errors [75,84,101]. However, other studies suggest that the DLPFC, which is responsible for the inhibition of reflexive saccades, is not involved in the additional processing required for mixed tasks [73].

Although the elevated AS error rate indicates a failure of inhibition, the prolonged correct response latency demonstrates that even when reflexive saccades are successfully inhibited, the process of generating an AS command remains slow. This may be related to damage to the frontal executive network and the BG [105]. Damage to the FEF may also be associated with prolonged AS latency [106]. Due to the involvement of the SEF, pre-supplementary motor area (pre-SMA), and ACC, PSP patients also lack the ability to monitor and correct saccadic errors [107]. In delayed AS tasks, this deficit in prefrontal working memory further reduces the proportion of correct responses, whereas the proportion of anticipatory prosaccades that cannot be suppressed during the delay period is abnormally elevated.

### 4.5. Predictive Saccades (PSs)

#### 4.5.1. Manifestations

In PS tasks, patients with PSP show decreased performance in both vertical and horizontal PS, with vertical impairment being more prominent [68].

#### 4.5.2. Pathophysiological Mechanisms of Abnormal Manifestations

The enrichment of 4R-tau proteins in the SEF, STN, cerebellar vermal cortex, and cerebellar efferent tracts in PSP leads to severe microstructural fiber demyelination and volumetric atrophy of the superior cerebellar peduncle (SCP). This disrupts internal timing prediction and rhythm synchronization mechanisms within the brain. Particularly for vertical PS, severe atrophy of the midbrain relay nuclei causes eye movements to degenerate into severely delayed reactive catch-up saccades that rely heavily on exogenous visual signals for activation.

### 4.6. Memory-Guided Saccades (MGSs)

#### 4.6.1. Manifestations

The oculomotor system plays a key role in maintaining spatial short-term memory (STM) and working memory. Studies show that compared with stimuli presented horizontally, PSP patients exhibit significant and highly specific impairment in STM along the vertical direction [62,63]. This impairment of spatial working memory is significantly more severe than PD [63]. In addition, the degree of impairment in PSP during feature search, where the target and distractors differ in only a single feature dimension, is significantly more severe than during conjunction search, where the target is defined by a combination of two or more independent features [63].

Unexpectedly, some PSP patients do not show absolute abnormalities when performing MGS tasks [61]. This may suggest that the autonomous initiation function of MGS, which reflects saccadic inhibitory control, is relatively preserved in the early stages of PSP. In follow-up studies, although the horizontal MGS latency in PSP patients at the early stages is comparable to that of HC, their accuracy and MGS success rate are significantly reduced [65]. As the disease progresses, the horizontal MGS accuracy in PSP undergoes continuous and significant deterioration, whereas PD patients show no such obvious change [65].

Regarding main sequence fitting, studies have shown that the velocity of small-amplitude saccades in PSP is largely normal, and significant velocity reduction occurs only in large-amplitude saccades. Therefore, the asymptotic velocity (*V*_0_) may be the most sensitive indicator for assessing advanced saccadic velocity abnormalities in PSP because it eliminates the influence of individual saccadic amplitudes [52]. This is also applicable to MGS tasks. However, when performing MGS tasks, PSP patients generally exhibit slower saccadic velocity and lower accuracy than during ProS tasks [85]. Interestingly, although horizontal MGS accuracy decreases in PSP, its amplitude varies significantly, while the peak velocity remains indistinguishable from HC [85].

#### 4.6.2. Pathophysiological Mechanisms of Abnormal Manifestations

The FEF, SEF, and PEF play important roles in the generation of spatial working memory [11]. Degeneration of the DLPFC and related prefrontal cortices in PSP patients leads to information blurring of the target spatial coordinates stored there [108]. When the delay period ends and the initiation command is issued, the spatial position signals transmitted from the cerebral cortex to the brainstem are inherently erroneous and variable, resulting in dispersion and error in the amplitude of the advanced saccades [109].

Because local circuits within the SC, particularly its superficial projection layers, bear the critical functions of constructing exogenous spatial salience maps and maintaining short-term spatial attentional orientation, pathological degeneration of SC neurons not only prevents PSP patients from maintaining stable vertical fixation but also directly cuts off the transient storage pathway for short-term spatial localization attention. This results in an inability to effectively complete position memory and feature retrieval for single-feature spatial targets, manifesting as a vertical-predominant spatial memory deficit. Because tau protein deposition in the midbrain oculomotor nuclei (riMLF, interstitial nucleus of Cajal (INC)) and the SC damages subcortical circuits responsible for exogenous attention and stimulus salience processing [62,63], patients are unable to rapidly and automatically detect targets with a single feature difference.

### 4.7. Others

Zhang et al. used a horizontal ProS Go/No-Go task combined with a hierarchical drift-diffusion model to reveal distinct mechanisms of response inhibition impairment between PSP and PD. Both PSP and PD exhibit a slowed rate of decision evidence accumulation and shortened non-decision time; however, the significant Go response bias unique to PSP explains the clinical paradox wherein severe oculomotor akinesia coexists with impulsive behavior. Saccadic parameters based on this computational model can effectively differentiate among PSP, PD, and healthy individuals, significantly outperforming raw behavioral metrics [110].

A small-sample study also investigated the remote distraction effect (RDE) task, where a green target appears on one side of the screen after the central fixation point disappears, and an irrelevant distractor is presented on the contralateral side in some trials, requiring participants to rapidly make a saccade to the target and ignore the distractor. Surprisingly, although the saccadic error rate toward distractors is significantly abnormal in PSP, the RDE in PSP is not abnormal [111]. Also, the RDE in PSP patients shows no clear association with frontal GABA abnormalities [111].

## 5. Differentiation of PSP Clinical Subtypes Using Advanced Saccadic Abnormalities

Differences exist in the quantitative comparison of ProS among clinical subtypes of PSP. One study found that the gain [22] and vertical peak velocity [76] in PSP-RS were significantly lower than those in PSP-P. The ProS peak velocity, accuracy, and SWJ incidence in all directions are more significantly abnormal in PSP-RS than in PSP-PGF [91]. Terao et al. suggested that MGS abnormalities are most prominent in the PSP-RS subtype [65]. Other studies have demonstrated that the degree of MGS and ProS abnormalities among different PSP subtypes exhibits a significant gradient: PSP-C > PSP-RS > PSP-P > PSP-PGF, and this gradient is more prominent in MGS [52].

However, some inconsistencies exist among study results. Some studies suggest that almost all advanced saccadic parameters show no significant differences between PSP-RS and PSP-P [25,64,65,74], such as peak velocity [22], latency [91], gain [76], and SI rate [76]. Nevertheless, it should be noted that PSP-RS progresses more rapidly than PSP-P; at 1 year from onset, oculomotor impairment in PSP-RS is already significantly more severe than in other subtypes, and the patterns of advanced saccadic abnormalities in other subtypes gradually converge toward PSP-RS as the disease progresses [91]. Consequently, the disease stages of patients with various PSP subtypes included in different studies differ.

The differences in advanced saccadic abnormalities among PSP subtypes stem from variations in the spatial distribution, chronological progression, and severity of tau protein deposition. In the PSP-RS subtype, extensive pathological damage occurs early in the midbrain tegmentum, riMLF, SC, and SCP, causing near-destructive disruption of the brainstem oculomotor center; thus, advanced saccadic abnormalities such as prolonged latency, decreased vertical saccadic velocity, and cognitively mediated oculomotor abnormalities appear early and are severe [20,112,113,114,115]. In the PSP-P subtype, tau protein is predominantly deposited in subcortical nuclei such as the striatum, STN, and globus pallidus (GP) during the first few years, while the midbrain burst center remains relatively intact, presenting only with mild upward vertical saccadic abnormalities and minimal overall oculomotor impairment. The PSP-C subtype features pathological involvement of cerebellar pathways as its core, with predictive and rhythm-related advanced oculomotor abnormalities being the most prominent. The PSP-PGF subtype exhibits the mildest involvement of the pontine tegmentum and the cerebellar dentate nucleus, with the least apparent advanced oculomotor abnormalities [5,116].

Additionally, the ProS may primarily depend on the parietal-SC reflex pathway, and its functional performance is largely constrained by brainstem burst centers such as the riMLF. The tau pathology of each PSP subtype exhibits a gradient not only in the brainstem but also in the invasion depth and propagation speed across micro-neural networks in multiple areas of the cerebral cortex. Therefore, MGS and AS tasks, which carry a relatively high cognitive load, are more capable of differentiating PSP subtypes than the ProS [11,117]. However, as the disease progresses, typically more than three years after PSP onset, differences in the pathological distribution among PSP subtypes gradually diminish [5], which may cause their advanced oculomotor abnormality patterns to converge.

## 6. Differential Diagnosis of PSP Using Advanced Saccadic Parameters

### 6.1. PD

Mean vertical ProS peak velocity may be the best indicator to differentiate PSP, especially PSP-P and PSP-RS, from PD [25,70,91], even at stages where they are clinically indistinguishable [22]. Additionally, individual studies have reported that vertical ProS amplitude has 100% sensitivity for PSP and that reduced vertical ProS peak velocity showed 99.1% specificity [17,70]. The composite metric, the product of AxV of ProS, has been proposed as a potentially useful VOG biomarker for differentiation across disease stages; in particular, downward saccade abnormalities have shown higher specificity for PSP than upward saccade abnormalities [17]. The comprehensive index proposed by Herwi et al., calculated as (vertical mean velocity/horizontal mean velocity) × (vertical gain/horizontal gain), also demonstrates certain advantages in differentiating early PSP from PD [79] (Table 4).

Horizontal AS peak velocity [61], horizontal ProS MQPV combined with visual fixation suppression (VS) [67], vertical ProS [70,72,79] and its dynamic parameters (power-function fitted values of peak velocity and duration) [26,93], horizontal GS gain [83], and horizontal AS error rate [83] can also assist in the differential diagnosis between PSP and PD. Observing whether patients exhibit a progressive decline in accuracy and peak velocity, known as saccadic fatigability, during a continuous single type of advanced saccadic task (GS or MGS) over a very short duration of 1 min can serve as a potential objective indicator to differentiate PSP from PD [85]. A follow-up study also found that a progressive decline in horizontal ProS and MGS accuracy can be used to distinguish PSP from PD [65]. Dale et al. reported that a camera-less computerized measurement of ProS latency differentiated mid-to-late-stage PSP-RS from PD-PIGD with 100% accuracy [89]. This estimate was derived from only five patients with PSP-RS and five patients with PD-PIGD and was not externally validated. It should therefore be interpreted as an exploratory association rather than as having definitive diagnostic accuracy. In conclusion, eye-tracking technology based on advanced saccades may help distinguish PSP from PD, particularly when standardized tasks are used [70].

### 6.2. MSA

Reductions in horizontal and vertical ProS peak velocities, particularly in the vertical direction [55,68], along with horizontal GS gain and AS error rates [83], can distinguish patients with PSP from those with PD and MSA. Although horizontal ProS parameters (latency, peak velocity, and accuracy) may overlap between PSP and MSA-P in the early stages [87], the peak velocity and gain of vertical ProS are significantly more severely impaired in PSP patients than in MSA [76]. Patients with degenerative Parkinsonism exhibit oblique saccade trajectory deviation, which is not due to a slowing of vertical saccadic velocity that causes a curved trajectory, but that the amplitude and gain of vertical saccades are significantly lower than those of horizontal saccades. The severity of oblique ProS trajectory abnormalities combined with vertical saccadic slowing serves as a characteristic feature assisting in differentiating PSP from PD, MSA, and CBD [72].

### 6.3. CBD

As previously mentioned, the distinct impairment patterns observed in mixed tasks alternating between ProS and AS can be used for the differential diagnosis of degenerative Parkinsonism. The most significant difference is that the directional error rate of PSP under ProS instructions is far lower than that of CBD patients [73]. Vertical EOG-measured peak velocities of ProS and AS [68], upward 10° mean saccade velocity [75], and the first gain of horizontal 10° OS [75] can also effectively differentiate PSP from CBD. Studies have shown that the horizontal GS peak velocity of patients with typical CBD remains normal throughout a 1-year follow-up, with a progressive prolongation of GS latency observed only on the side dominant for apraxia symptoms [84]. This clearly demonstrates that EOG can improve the accuracy of early differentiation between PSP and typical CBD. The dissociation phenomenon of horizontal GS may be the key discriminative feature between PSP and CBD [83]. In the early stages, PSP presents with hypometric horizontal GS amplitude but normal latency, whereas early CBD presents with a significantly prolonged horizontal GS latency but entirely normal amplitude. Additionally, some patients with atypical CBD who are subsequently diagnosed with PSP after follow-up can exhibit abnormal GS and AS manifestations similar to PSP during early evaluations. Horizontal EOG can establish a diagnosis 4–6 months earlier than clinical diagnosis [84].

### 6.4. AD

The impairment of the first gain of horizontal and vertical OS in patients with PSP is far more severe than in patients with AD, holding promise for rapid, non-invasive differentiation between the two conditions [75]. The upward 10° mean saccade velocity demonstrated an AUC of 1.0 for differentiating PSP from AD [75]. This apparently perfect result should be interpreted cautiously because small samples and restricted disease spectra can inflate diagnostic performance, and the findings have not been confirmed in an independent external cohort. Notably, typical sporadic AD most often presents with early and prominent episodic memory impairment. In such cases, the clinical phenotype is usually distinct from PSP, and oculography is not generally required solely to establish an AD diagnosis [118,119]. By contrast, atypical sporadic AD may begin with predominant visuospatial, language, executive, behavioral, or motor deficits rather than episodic memory impairment, which can delay recognition and increase diagnostic uncertainty [118,119]. In these presentations, cerebrospinal fluid (CSF) Aβ42/40 and phosphorylated tau (p-tau) or amyloid/tau positron emission tomography (PET) can provide biological confirmation, although lumbar puncture and limited access to molecular imaging can restrict their routine use [119,120]. Quantitative oculography may therefore provide a rapid, non-invasive adjunct when the AD phenotype is atypical or the differential diagnosis remains uncertain [121].

### 6.5. Others

Delayed AS tasks can be used to distinguish frontotemporal dementia (FTD) from PSP [103]. The accuracy, velocity, and latency of ProS, along with more frequent SWJs, show the most significant differences between patients with anti-IgLON5 disease and PSP and can also effectively differentiate both diseases from HC [74]. X-linked dystonia-Parkinsonism (XDP) can be distinguished from PSP by the normal vertical OS peak velocity, along with more severe low horizontal OS gain, prolonged vertical OS latency, dual deficits in both latency and amplitude of MGS, and a high AS error rate in patients with XDP [122].

### 6.6. Mechanisms of Differentiating PSP from Other Diseases via Advanced Saccades

During the early course of the disease, the descending frontal fibers to the BG and the prefrontal-caudate pathway are functionally impaired to varying degrees in PD and PSP, causing horizontal advanced saccades in both conditions to exhibit deficits induced by attention deficits and dopaminergic dysfunction [123]. Due to differences in the pathology of brainstem burst neurons, reductions in vertical ProS velocity and amplitude serve as candidate biomarkers distinguishing PSP from PD. Unlike in PSP, the brainstem burst neurons in early PD are largely intact [101], so the peak velocity and latency of its reflexive ProS may not be significantly abnormal. Saccadic abnormalities in PD mostly originate from the loss of dopaminergic neurons in the SN, leading to excessive tonic inhibition of the SC by the BG [101]. Consequently, PD manifests low-gain hypometria in voluntary saccades and MGS.

Although PSP also involves degeneration of the SN, PSP features widespread neurodegeneration in cortical and subcortical nuclei, and the incidence of STN atrophy is significantly higher than in PD [67]. Saccadic fatigability, manifested as a progressive failure of burst capability during consecutive MGS or GS tasks over a short period in PSP, can also be differentiated from the constantly low-gain saccades in PD. CBD patients have deficits in motor planning and spatial attention allocation (oculomotor apraxia), due to asymmetric frontoparietal cortical (FEF/PEF) involvement, rather than damage to the brainstem saccade generator circuitry [84,124,125]. The cerebral cortical control areas in PSP may be relatively spared in the early stages. Distinct from the “round-the-houses” phenomenon during ProS execution in PSP, MSA differs significantly due to prominent SWJs and central nystagmus resulting from damage to the vestibulocerebellar system [126,127]. In AD, amyloidosis damages advanced cognitive circuits including the DLPFC, causing impairment in AS response inhibition and error correction capability, while their midbrain and pontine saccadic burst generators remain intact [42,101]. Due to extensive degeneration of the midbrain tegmentum in patients with PSP, the first gain of OS is significantly more severely impaired than in AD, potentially serving as a candidate biomarker for rapid, non-invasive differentiation between the two conditions.

## 7. Correlation Between Advanced Saccadic Parameters and Clinical Functional Indices in PSP

Advanced saccadic parameters are not isolated neuroelectrophysiological manifestations. Instead, they are closely associated with disease severity, motor deficits, and cognitive and behavioral impairment of PSP, objectively reflecting overall disease progression and clinical phenotype severity. Multiple studies have analyzed correlations among disease scale scores, cognitive function, and oculomotor indices, establishing relationships between quantitative oculomotor parameters and clinical phenotypes. However, current findings exhibit some heterogeneity.

Some studies suggest that horizontal saccadic abnormalities in PSP increase the risk of falls by impairing rapid visual focusing, PS, and convergence responses [128]. Conversely, vertical gaze palsy, a core early feature of PSP, cannot differentiate between high and low fall risk because it is already present in most patients at diagnosis. The accuracy [65] and peak velocity [52] of horizontal ProS and MGS are significantly and negatively correlated with HY stage, whereas ProS latency shows a weak positive correlation with HY stage [65]. Interestingly, the acceleration and deceleration phases of horizontal ProS, as well as the deceleration phase of horizontal MGS, correlate significantly and positively with HY stage. This suggests that advanced saccadic movements become more sluggish and less efficient in later stages of the disease. Notably, the correlation between MGS and disease stage exhibits a distinct spatial eccentricity specificity, presenting a significant positive correlation with HY stage only during the task with a large eccentricity of 30° [52]. Some studies have failed to observe statistical associations between disease duration or HY stage and various oculomotor parameters. This heterogeneity may stem from differences in disease duration stratification, subtype composition, and medication status among enrolled patients [22,66,87,91]. A small-sample study demonstrated that the Mini-Mental State Examination (MMSE) score in PSP does not correlate with core parameters of horizontal or vertical ProS and AS [90], indicating that basic global cognitive scores may have limited matching capacity with fine oculomotor circuit damage.

At the level of functional scale scores, advanced oculomotor parameters accurately correspond to multidimensional deficits in motor and executive functions in PSP. Frontal behavioral function is highly correlated with oculomotor indices of inhibitory control [129]. Specifically, shorter horizontal GS latencies and higher AS error rates are linked to more severe frontal behavioral dysfunction [83,86]. Horizontal ProS latency correlates significantly and positively with Progressive Supranuclear Palsy Rating Scale (PSPRS) scores and significantly negatively with Frontal Assessment Battery (FAB) scores [25]. Vertical ProS peak velocity shows a significant negative correlation with PSPRS scores and significant positive correlations with FAB and Montreal Cognitive Assessment (MoCA) scores [25]. These findings confirm that more severe advanced oculomotor impairment indicates poorer motor and frontal cognitive functions in PSP. The associations between advanced oculomotor parameters and clinical symptoms display marked symptom specificity. Vertical ProS peak velocity, latency, and accuracy correlate significantly with the dysphagia subscore of the PSPRS. More pronounced advanced oculomotor deficits are linked to more severe dysphagia, yet show no significant relationship with the postural instability subscore [91]. This suggests that damage to the brainstem oculomotor center is highly synchronized with the degeneration of brainstem swallowing pathways, whereas postural instability may involve an independent pathological regulatory pathway. Additionally, Unified Parkinson’s Disease Rating Scale (UPDRS) scores show no significant correlation with horizontal or vertical ProS peak velocity or gain [66]. General Parkinsonian motor scales may not adequately reflect PSP-specific brainstem oculomotor dysfunction, further highlighting the value of advanced saccadic parameters for the assessment of PSP.

## 8. Correlation of Advanced Saccadic Parameters with Neuroimaging and Brain Functional Metabolism

The neuroimaging basis of advanced saccadic abnormalities involves structural atrophy, microstructural damage, and reduced brain metabolism within central oculomotor regulatory circuits [25,76,130]. Quantitative advanced saccadic parameters have been shown to correlate with structural, metabolic, and microstructural alterations in oculomotor-related regions, including the midbrain, SCP, brainstem pathways, and cortical networks [17,55,76,88]. These findings support the anatomical and pathological basis of oculomotor abnormalities in PSP from an imaging perspective and provide neuroimaging evidence for the neural mechanisms underlying oculomotor biomarkers. Importantly, these associations should be interpreted as reflecting shared neurodegenerative processes rather than indicating that oculomotor abnormalities themselves alter neuroimaging measurements.

### 8.1. Correlations with Structural Neuroimaging

Midbrain structural atrophy is the most central neuroimaging feature of PSP and serves as the primary anatomical basis for vertical advanced saccadic abnormalities. Multiple studies in patients with PSP consistently confirm that vertical ProS peak velocity correlates significantly and positively with midbrain volume and the midbrain tegmental planar area [76]. Specifically, more severe midbrain atrophy leads to a more pronounced reduction in vertical ProS velocity. Vertical ProS amplitude, peak velocity, and their product are positively correlated with midbrain area and negatively correlated with quantitative indices of midbrain atrophy, such as the Magnetic Resonance Parkinsonism Index (MRPI) and MRPI 2.0, thereby precisely reflecting the degree of midbrain neurodegeneration [17]. The vertical ProS gain and a novel diagnostic index of PSP, defined as (vertical mean velocity/horizontal mean velocity) × (vertical gain/horizontal gain), are also significantly and positively correlated with midbrain volume [79].

Beyond the midbrain regions, studies in patients with PSP have shown that advanced saccadic parameters are associated with structural alterations in the cerebellum, cerebral cortex, and white matter. Vertical ProS peak velocity and accuracy significantly correlate with SCP volume, confirming the critical role of cerebellar efferent pathways in vertical saccade regulation [91]. Prolonged ProS latency is associated with cerebral white matter alterations, particularly parietal white matter atrophy [76]. Concurrently, smaller cortical volumes in the bilateral parietal, occipital, and temporal lobes are associated with more pronounced ProS latency prolongation in PSP [75]. Notably, the characteristic “multi-step pattern” and low-amplitude saccades of PSP show no clear correlation with localized brain region volumes [76]. This suggests that such subtle oculomotor abnormalities in PSP may not result from isolated regional atrophy but may reflect dysfunction within distributed neural networks and disrupted circuit coordination across multiple brain regions.

### 8.2. Correlations with Metabolism and Diffusion Tensor Imaging (DTI)

PET studies in patients with PSP further demonstrate that advanced saccadic parameter abnormalities are associated with hypometabolism in the cortex and nuclei of central oculomotor circuits. Prolonged horizontal ProS latency significantly correlates with reduced glucose metabolism in the bilateral PPC and precuneus [88]. The decrease in horizontal ProS mean velocity in PSP corresponds to hypometabolism in the left SC and dorsal pontine nucleus, whereas reduced vertical ProS gain is closely associated with hypometabolism in the right SC [88]. These findings suggest functional differences in the involvement of hemispheric and subcortical regions within horizontal and vertical advanced saccadic pathways. Notably, AS error rates in patients with PSP show no specific association with frontal cortex metabolism [88]. This further supports that saccadic inhibition deficits in PSP do not stem solely from frontal metabolic damage but result from coordinated multi-circuit abnormalities. Additionally, the functional integrity of the cerebellum and lower-level visual cortex forms an essential foundation for safeguarding saccadic motion efficiency. Studies have revealed that the maximum peak velocity of horizontal ProS in PSP exhibits a significant positive correlation with glucose metabolism in the right rostral cerebellar vermis (lobule IV/V, culmen) and the right lingual gyrus (area V3) [78].

DTI reveals mechanisms of oculomotor impairment based on the microstructural integrity of brain regions. The horizontal ProS peak velocity in PSP correlates significantly and positively with the fractional anisotropy (FA) values of regions including the pons, SCP, and medial lemniscus (ML), whereas vertical ProS peak velocity primarily depends on the microstructural integrity of the midbrain and pontine crossing tract (PCT) [55]. Lower FA values in these pathways indicate more severe nerve fiber demyelination and axonal injury, leading to more pronounced abnormalities in saccadic velocity, which provides a microstructural explanation for the advanced saccadic kinematic deficits in PSP.

### 8.3. Correlations with Functional Magnetic Resonance Imaging (fMRI) Activation Pattern

The analysis of blood oxygen level-dependent (BOLD) signals shows that the PSP-RS subtype exhibits significant task-induced hypoactivation and impaired default mode network (DMN) deactivation. Furthermore, the damage patterns across brain regions differ markedly between saccadic paradigms and directions, precisely corresponding to clinical oculomotor phenotypes. When performing horizontal and vertical ProS, patients with PSP-RS display significantly reduced BOLD activation in frontostriatal networks, including the FEF, SEF, CN, and thalamus, as well as the occipital visual cortex, alongside insufficient deactivation of the DMN, which includes the orbitofrontal cortex, insula, and inferior parietal lobule (IPL). Among these, brain functional impairment is more extensive during vertical ProS, which additionally features decreased activation in the fusiform gyrus, posterior cerebellar lobe, and cerebellar uvula [90]. Unlike HC, PSP-RS patients require more intense DMN deactivation, involving the right middle temporal gyrus, superior temporal gyrus, and IPL, for vertical ProS than for horizontal ProS, further underscoring the central vulnerability of the vertical oculomotor circuit [90].

During AS cognitive inhibition tasks, the brain injury patterns split significantly between horizontal and vertical saccades. Horizontal AS only exhibits reduced activation in the right middle occipital gyrus and insufficient DMN deactivation in the dorsomedial prefrontal cortex (dmPFC), posterior cingulate cortex, and parahippocampal gyrus, with no significant differences in frontostriatal circuit. In contrast, vertical AS presents significantly decreased activation in frontostriatal network, including the FEF, SEF, putamen, and thalamus, as well as the posterior cerebellar lobe and cerebellar nodulus, accompanied by more widespread insufficient DMN deactivation [90]. This likely indicates that cognitive inhibitory saccades in the vertical direction are more sensitive to damage within cortical–BG–cerebellar circuits. Notably, current fMRI studies cannot separate BOLD signals induced by implicit attentional regulation from those caused by eye movements themselves. Interpretations of these differences in brain activation must remain cautious and should be comprehensively validated alongside structural and metabolic neuroimaging features.

## 9. Limitations and Open Questions

### 9.1. Discrepancies in Research Findings

Whether these advanced saccadic parameters can achieve a differential diagnosis of PSP remains controversial. Linder et al. argued that in the early stages of the disease, ProS parameters cannot distinguish PSP from PD and MSA-P [87]. Other studies suggest that ProS and AS detected by EOG might differentiate early PSP from PD, MSA, CBD, and vascular Parkinsonism (VP). Nevertheless, due to its limited specificity, EOG alone is not recommended as the sole neurophysiological examination for early APS [68]. Some early studies utilized the National Institute of Neurological Disorders and Stroke and Society for PSP (NINDS-SPSP) criteria, which required the presence of visible vertical supranuclear gaze palsy or severe early backward falls. This automatically excluded many patients in the very early or so-PSP stages, as well as those with atypical PSP subtypes whose oculomotor abnormalities were not yet prominent, from the inclusion criteria. In contrast, recent studies mostly follow the 2017 Movement Disorder Society (MDS) diagnostic criteria proposed by Höglinger et al. This standard incorporates the staging of oculomotor disorders, enabling the inclusion of patients in the subclinical phase and those with mild atypical subtypes. Accordingly, studies applying these two diagnostic criteria present biases in the overall pathological distribution and severity of the samples, which may confound the statistical similarities and differences among various oculomotor parameters.

Furthermore, individual variation in latency is large among patients with early PSP [80,83]. Notably, an early study using EOG that strictly enrolled patients with similar disease durations demonstrated that, despite the progressive slowing of horizontal GS velocity, patients experienced a phase where latency regressed toward the normal mean before prolonging again over a 1-year follow-up period [86]. This phenomenon may occur because shortened latency stems from severe impairment of the frontal inhibitory system, whereas prolonged latency results from damage to excitatory subcortical pathways, such as the SC, or visual attention-shifting circuits. In the early stage of PSP, either the inhibitory or the excitatory pathway exhibits more severe impairment. As the disease progresses and the degree of damage to these two pathways becomes balanced, the inter-individual dispersion of latency decreases, and latency reaches an equilibrium. In late-stage PSP, multiple excitatory pathways are simultaneously impaired, leaving saccade initiation uncompensated, which leads to a secondary prolongation of latency [86]. Similarly, Ghosh et al. found that horizontal ProS latency progressed slowly within 1 year. However, they concluded that the annual rate of change in latency was small, making it unsuitable as a biomarker for 1-year disease progression in PSP [80].

Variations in oculomotor testing protocols also influence results. When determining the onset of a saccade, utilizing different instantaneous velocities as the saccadic threshold or directly applying acceleration thresholds can alter the calculated latency and duration [131]. Schneider et al. also found that when the eyes of patients with PSP were positioned in the upper visual field, the degree of slowing and hypometria in downward saccades was significantly greater than that in upward saccades [71]. Therefore, future oculomotor studies must carefully control the initial position of the eyes within the orbit.

### 9.2. Limitations of Reported Diagnostic Performance

Several studies reported high sensitivity or specificity (Table 4), an AUC close to 1.0 [17,70,75], or 100% diagnostic accuracy [89]. An AUC of 1.0 indicates perfect discrimination, values between 0.70 and 0.90 suggest potentially useful diagnostic accuracy, whereas an AUC of 0.5 indicates no discriminative ability and values below 0.5 indicate performance worse than chance. However, these estimates were derived from small and selected cohorts and may therefore be affected by small-sample bias and substantial statistical uncertainty. Spectrum bias is also possible because studies often included clinically established or probable PSP-RS and compared them with clearly defined control groups, whereas early and variant PSP phenotypes are more difficult to distinguish in routine clinical practice. This may lead to overestimation of diagnostic performance.

Overfitting may occur when multiple oculomotor variables or cut-off values are explored and the best-performing result is evaluated in the same dataset. In addition, most reported thresholds or models have not been tested in independent cohorts, so their generalizability remains uncertain. Accordingly, perfect or near-perfect diagnostic estimates should be regarded as preliminary and require confirmation in adequately sized, clinically representative cohorts with external validation.

### 9.3. Potential Reasons for the Mismatch Between Neuroimaging Changes and Advanced Saccadic Abnormalities

Structural MRI measures cumulative morphological changes at the end stage, characterized by substantial neuronal loss and gliosis [112]. In contrast, advanced oculomotor parameters serve as sensitive central functional electrophysiological indices. Their normal execution depends not only on neuronal count but also strictly requires synaptic signal transduction, neurotransmitter homeostasis, and synchronized neural network discharge [21]. In the early stages of PSP, presynaptic accumulation of 4R-tau oligomers, axonal transport deficits, acetylcholine depletion, and localized electrical desynchronization have already severely impaired the function of burst neurons in the riMLF or descending inhibitory pathways from the prefrontal cortex. This may cause oculomotor indices to deteriorate before morphological abnormalities become apparent on MRI. Furthermore, when moderate tau pathology affects subcortical centers such as the BG and midbrain SC, compensatory hyperactivation of the PEF cortex and profound deactivation of the DMN can mobilize additional attentional and cognitive control resources. This mechanism allows indicators such as MGS gain and AS error rates to remain near-normal levels for a certain period, thereby masking existing subcortical structural atrophy until the compensatory network completely collapses in late stages.

### 9.4. Practical Limitations of Clinical Implementation

Clinical implementation may also have practical constraints. Acquisition time depends on the protocol. A targeted mobile fixation paradigm can be completed in less than 20 s [70], whereas a comprehensive VOG assessment requires calibration, repeated trials, artifact review, and data analysis, so the total visit may take 10 to 15 min or longer [21]. High-end clinical-grade oculography systems commonly used in clinical studies and specialist clinics, such as EyeSeeCam and ICS Impulse [132], can cost approximately US$40,000. Dedicated VOG requires hardware, calibration, trained personnel, data-quality checks, and analysis time, but portable eye trackers, HMD, smartphones, and tablets may reduce equipment burden and improve scalability [133]. However, tracking accuracy and sampling rate vary widely across these consumer-grade devices, and their equivalence to dedicated VOG systems for vertical saccadic measurement has not been established. Recent research described these digital approaches as potentially cost-effective, while noting that formal cost-effectiveness and real-world implementation evidence remain limited in diagnosing neurodegenerative diseases [19]. Notably, in a multicenter retrospective study enrolling 120 patients with vestibular symptoms, Ni et al. found that, based on Medicare reimbursement rates, the cost per VNG with abnormal findings was $869.57, whereas the cost per VNG leading to a change in both diagnosis and treatment was $5454.55 [134]. Additionally, access to quantitative oculography is currently largely restricted to tertiary referral centers with dedicated vestibular function centers or movement-disorder clinics. Advanced oculomotor paradigms for PSP, such as AS and MGS tasks, are not routinely performed in general neurology or community settings. PSP-specific waiting-time data for tertiary oculography services have not been reported, so no generalizable estimate can be provided. Access depends on local equipment, staffing, and referral pathways.

## 10. Conclusions and Perspectives

Traditional bedside oculomotor assessment relies heavily on the subjective experience and manual judgment of clinicians, presenting significant limitations in standardization and detection accuracy. Verbal instructions, visual targets, and the distance to the patient’s primary gaze position are difficult to standardize completely across different physicians, and the observation of downward saccades is often obstructed by the natural drooping of the upper eyelid. Quantitative oculography can provide important additional information when PSP is suspected but oculomotor signs remain subtle or equivocal, particularly early in the disease and in PSP-P, when differentiation from PD may still be difficult. Clinical examination is less accurate than quantitative oculography for identifying pathological saccadic slowing, even for clinicians who have undertaken subspecialty neuro-ophthalmology fellowship training following core neurology residency [135]. The PSP cases in that study had a disease duration of 1 to 2 years, and the broader patient sample included individuals with a full or only mildly reduced range of gaze despite objectively slow saccades. This supports quantitative testing before a clear vertical supranuclear gaze palsy becomes evident. When PSP is accompanied by prolonged latency or saccadic hypometria, the velocity of eye movements cannot be accurately judged by the naked eye alone. More importantly, these signs are not specific to PSP and can also occur in healthy older adults and PD patients, further increasing the difficulty of subjective clinical discrimination [64].

The MDS PSP criteria recognize slow vertical saccades as a core oculomotor feature and allow quantitative measurement of saccadic velocity, although they do not mandate a specific protocol or universal cut-off [20]. Oculography is therefore most useful after the initial neurological examination when APS remains diagnostically uncertain. If the first recording is nondiagnostic but suspicion persists, repeat testing can be considered as the phenotype evolves, although no fixed evidence-based interval has been established. Once vertical supranuclear gaze palsy is clinically clear, quantitative recording may contribute more to objective phenotyping and longitudinal assessment. Overall, oculography is best positioned as an important adjunct that can strengthen earlier and more objective recognition of PSP.

Horizontal and vertical ProS should remain the core quantitative assessment because peak velocity, amplitude, and gain can be measured with relatively low cognitive demand. MGSs depend on attention and spatial working memory, ASs require response inhibition, and PSs additionally engage temporal expectation [136]. It should be noted that these higher-order tasks such as MGSs are relatively resistant to age-related physiological oculomotor degeneration, avoiding confounding interference caused by natural aging in the elderly population [85]. Higher-order tasks can then be added when cognitive or executive dysfunction is clinically relevant or when broader imaging features may help resolve diagnostic uncertainty. Their interpretation should account for task comprehension, visual acuity, alertness, fatigue, and calibration quality. Repeated trials, predefined quality-control criteria, age-appropriate reference data, and consistent testing conditions can reduce individual variability. Within-subject measures, such as vertical-to-horizontal velocity or gain ratios, may further improve interpretability by using each PSP patient as an internal reference. Thus, advanced eye movement tasks are most useful as complementary phenotyping tools rather than isolated disease-specific markers [137].

Current research on advanced oculomotor function in PSP remains limited by predominantly single-center designs, small sample sizes, and differences in cohort composition and experimental implementation. Although several core task parameters and conventional saccadic measures, including target amplitude, stimulus conditions, and the definition or calculation of peak velocity, are generally well established, other methodological details are not consistently reported or applied across studies. These include medication status during testing, sampling frequency, calibration procedures, criteria for identifying saccade onset and offset, data preprocessing, and quality-control procedures. Such differences may reduce reproducibility and limit direct comparisons between studies [138]. Nevertheless, independent studies converge on disproportionate vertical saccadic slowing and hypometria as clinically informative features of PSP. Measures combining amplitude and peak velocity, as well as vertical-to-horizontal velocity and gain indices derived from quantitative oculography, have shown promise for distinguishing PSP from PD in the early stages of disease. Reduced peak velocity may also support differentiation from MSA, while the gain-latency pattern may help distinguish PSP from CBD. Therefore, a pragmatic clinical pathway is to quantify vertical and horizontal ProS first and add MGS, AS, or PS paradigms when they address a specific diagnostic or cognitive question. Incorporation into movement-disorder neuro-ophthalmology services is particularly relevant for early or diagnostically uncertain APS, where quantitative results that are determined non-invasively and by considering accessibility may add meaningful information to bedside assessment. With further standardization and device-specific validation, oculography could become an increasingly accessible component of early PSP assessment and longitudinal monitoring.

The current evidence nonetheless supports quantitative advanced saccadic features as promising candidate biomarkers rather than merely descriptive ocular motor abnormalities. However, the rarity and rapid progression of PSP, together with travel difficulties and the burden of eye movement testing, make recruitment into large prospective cohorts challenging. Multicenter studies with harmonized acquisition and analysis workflows, stage-stratified cohorts, and prospective external validation are needed to define transferable thresholds, false-positive rates, and predictive values. Establishing a standardized PSP oculomotor database would support external validation and provide a stronger basis for the clinical practice of advanced oculomotor indicators.

A single oculomotor parameter inherently lacks sufficient sensitivity to comprehensively cover the complex pathology and clinical phenotypes of PSP. Therefore, the direction of future research lies in constructing a multidimensional and multimodal biomarker fusion system. Vertical saccadic impairment in PSP patients may be significantly associated with the quality and quantity of both non-rapid eye movement (NREM) and rapid eye movement (REM) sleep [96]. However, the intrinsic link between advanced oculomotor function and sleep disturbances remains unclear and represents a critical avenue for future mechanistic research. Future approaches can transcend the traditional single laboratory paradigm by introducing multidimensional oculomotor tasks in real-world scenarios [139] to comprehensively capture the dynamic oculomotor functional deficits of PSP patients. Advanced quantitative oculomotor parameters should be combined with wearable digital indicators such as gait kinematics, speech acoustic characteristics, facial microexpressions, fine motor functions, and sleep rhythms [140,141,142]. Concurrently, fluid pathological biomarkers, including peripheral blood p-tau, neurofilament light chain (NfL), and CSF amyloid-beta (Aβ) and tau [143], can be integrated to multidimensionally characterize the features of PSP from the perspectives of macroscopic functional phenotypes to microscopic neural network damage and molecular pathology [144]. This multimodal fusion strategy not only helps differentiate PSP from other degenerative Parkinsonian syndromes at early stages but also lays a critical foundation for constructing individualized disease progression prediction models and remote monitoring systems.

## Figures and Tables

**Table 1:** Comparison of characteristics of different clinical eye movement recording methods.

	VOG/VNG	EOG/ENG	Infrared	Search Coil
Vertical recording	Good; capable of observing and recording torsional eye movements	Good; capable of observing and recording torsional eye movements, but easily affected by blinking	Affected by blinking	Good; capable of recording torsional eye movements
Preparation	Fast; requires calibration; unaffected by corneo-retinal potential	Slow; requires calibration; affected by corneo-retinal potential	Laborious adjustment; requires aligning the infrared emitter and receiver with the iris and sclera	Slow; requires calibration

**Table 2:** Summary of six advanced saccade tasks.

Task	Definition	Primary Brain Regions Involved	Cognitive Domain and Function
ProS	Subjects fixate on a central point and rapidly shift their gaze to a new peripheral visual target that appears without warning.	PFC, FEF, SEF, PEF	Guidance of visual attention, spatial orientation, basic motor execution.
AS	Subjects fixate on a central point. When a unilateral peripheral stimulus appears, they must inhibit the reflexive impulse to look at it and instead initiate a voluntary saccade to the mirror-symmetrical opposite position.	FEF, SEF, DLPFC, PEF, ACC, CEF	Control of reflexive impulses and voluntary inhibition, reflecting prefrontal cortex network integrity.
MGS	While subjects fixate on a central point, a peripheral target flashes briefly and disappears. Subjects maintain central fixation during a delay period and initiate a saccade based on memorized location information once the central point is extinguished.	FEF, SEF, PEF, DLPFC, PPC	Maintenance and retrieval of spatial short-term memory, and active working memory.
PS	Subjects track a target that alternates regularly at constant time intervals between specific spatial locations (e.g., left and right). After several practice trials, subjects initiate a saccade before the target actually moves.	FEF, SEF, DLPFC, Ce	Internal temporal modeling, rhythmic synchrony, cerebello-cerebral cortical integration.
GS	The central fixation point is extinguished before the peripheral target is illuminated, inducing a rapid saccade after this gap period.	FEF, PEF, caudal SC	Ability to actively disengage visual attention.
OS	The central fixation point remains illuminated when the peripheral target appears, requiring subjects to shift attention and initiate a saccade in the presence of competing visual stimuli.	FEF, PEF, rostral SC	Allocation mechanism of visual attention and voluntary inhibition of the existing fixation target.

DLPFC, dorsolateral prefrontal cortex; ACC, anterior cingulate cortex; PPC, posterior parietal cortex; SEF, supplementary eye field; FEF, frontal eye field; PEF, parietal eye field; CEF, cerebellar eye field; Ce, cerebellum; SC, superior colliculus.

**Table 3:** Studies of advanced saccade examinations in PSP patients.

Study	Year	Sample	Measurement	Mean Age yrs	Mean Disease Duration yrs	Task	Key Findings
[61]	2025	PSP (*n* = 47), PD (*n* = 12), HC (*n* = 18)	VEOG	75.6	4.8	ProS, AS, MGS	Prob+poss-PSP and so-PSP showed significant AS abnormalities compared with HC; horizontal AS peak velocity was significantly lower in so-PSP than in PD and HC. Most so-PSP patients with normal baseline AS parameters were reclassified as PD after follow-up.
[62]	2018	PSP (*n* = 8), HC (*n* = 8)	EOG	70	NA	ProS, MGS	7/7 PSP with complete absence of vertical ProS, 6/7 PSP with abnormal main sequence and restricted horizontal oculomotor range. Vertical Corsi span was significantly lower than horizontal Corsi span.
[63]	2021	PSP (*n* = 8), PD (*n* = 9), HC (*n* = 11)	EOG	69.5	2.9	ProS, MGS	Vertical saccades completely absent in PSP. Horizontal amplitudes significantly lower than HC; leftward also lower than PD.
[17]	2022	PSP-RS (*n* = 31), PSP-P (*n* = 22), PSP-PGF (*n* = 3), PD (*n* = 113), HC (*n* = 40)	VOG	71.2	3.8	ProS	PSP showed significantly reduced amplitude, peak velocity, and AxV of vertical ProS compared to PD and HC.
[64]	2021	PSP-RS (*n* = 55), PSP-P (*n* = 42), HC (*n* = 49)	VOG	71	3	ProS	Vertical saccade peak velocity and SI rate together identified approximately 80% of PSP.
[65]	2013	PSP-RS (*n* = 22), PSP-P (*n* = 6), PSP-C (*n* = 6), PSP-PGF (*n* = 2), PD (*n* = 66), HC (*n* = 58)	EOG	69.0	1–8	ProS, MGS	Horizontal saccade parameters can be used to differentiate PSP from PD and monitor disease progression.
[66]	2025	PSP (*n* = 50), HC (*n* = 50)	VOG	71	3	ProS	Joint examination of vertical ProS and SIFS significantly improves the discrimination between PSP and HC.
[67]	2023	PSP-RS (*n* = 11), PSP-PGF (*n* = 6), PSP-P (*n* = 5), PSP-C (*n* = 4), PD (*n* = 26), HC (*n* = 26)	ENG	74.2	4.4	ProS	Combined detection of VS and horizontal saccade velocity can assist in the differential diagnosis between PSP and PD.
[68]	2003	PSP (*n* = 14), PD (*n* = 6), MSA (*n* = 12), CBD (*n* = 6), VP (*n* = 3)	EOG	72.5	1.8	ProS, PS	EOG has predictive diagnostic value for early PSP in atypical Parkinsonism.
[69]	2019	prob-PSP (*n* = 16), HC (*n* = 17)	VOG	72	3.6	ProS	Vertical ProSs were more severely affected than horizontal ones.
[70]	2012	poss/pro-PSP (*n* = 10), PD (*n* = 11), HC (*n* = 10)	VOG	65.9	3.9	ProS	Combined analysis of peak velocity and amplitude via the <20 s fixation protocol is a reliable tool to differentiate PSP from PD and HC.
[71]	2011	prob-PSP (*n* = 7), PSP-P (*n* = 1), HC (*n* = 5)	magnetic search coil	73.5	3	ProS	Downward ProS amplitude was significantly smaller in PSP than HC when the head was pitched forward/eyes were in the upper field.
[72]	1996	PSP (*n* = 4), PD (*n* = 5), MSA (*n* = 8), CBD (*n* = 3)	magnetic search coil	68.3	6.5	ProS	Measurements of the gain and trajectory of oblique ProS may aid in diagnosing different types of Parkinsonism.
[73]	2007	PSP (*n* = 12), PD (*n* = 15), CBD (*n* = 8), HC (*n* = 10)	EOG	66	2.7	GS, AS	The paradigm of interleaved ProS and AS may increase the accuracy of differential diagnosis between PSP and CBD.
[74]	2021	PSP-RS (*n* = 5), PSP-P (*n* = 5), HC (*n* = 10)	VOG	69	2	ProS	Accuracy and peak velocity were significantly lower than those of HC, significantly prolonged latency; 9/10 PSP with SWJs.
[75]	2008	HC (*n* = 27), Prob-PSP (*n* = 10), CBS (*n* = 15), AD (*n* = 28)	dual Purkinje image eye tracker (DPI)	65.5	3.3	GS, OS, AS	Oculomotor assessment, particularly of vertical ProS and horizontal AS, is superior to traditional neuropsychological tests in differentiating PSP.
[76]	2017	PSP-P (*n* = 18), PSP-RS (*n* = 14), PD (*n* = 39), MSA-P (*n* = 13), MSA-C (*n* = 5), HC (*n* = 24)	VOG	72	3	ProS	The characteristic vertical peak velocity impairment in PSP is closely associated with midbrain atrophy.
[77]	1979	PSP (*n* = 9), HC (*n* = 4)	EOG	57.2	1.5–6	ProS	Clinically observed “slow saccades” included saccades with reduced peak velocity and saccades composed of a series of small, consecutive fractionated saccades.
[55]	2017	PSP (*n* = 30), PD (*n* = 36), MSA (*n* = 18), HC (*n* = 23)	VOG	71	3	ProS	Characteristic saccadic slowing in PSP is specifically associated with microstructural damage in brainstem and cerebellar pathways.
[78]	2014	PSP (*n* = 26)	EOG	71.3	2.8	ProS	Horizontal ProS abnormalities were associated with dysfunction of the rostral cerebellar vermis.
[79]	2021	prob-PSP (*n* = 8), poss-PSP (*n* = 5), PD (*n* = 15), HC (*n* = 16)	head-mounted display (HMD)	71.9	2.4	ProS	(Vertical Velocity/Horizontal Velocity) × (Vertical Gain/Horizontal Gain) for differentiating PSP from PD
[80]	2013	poss-PSP-RS and prob-PSP-RS (*n* = 23), HC (*n* = 22)	infrared sclerometry	71.1	3.0	ProS	The latency of horizontal ProS is significantly prolonged in PSP and progresses slowly over 1 year.
[81]	1982	PSP (*n* = 4), PD (*n* = 4), HC (*n* = 4)	infrared reflection oculography (IROG)	61.0	2–5	OS	PSP took significantly longer than HC and PD to complete all visual search and scanning tasks.
[82]	2017	PSP (*n* = 12), HC (*n* = 4)	limbus tracker	50–74	5.9	ProS	Both horizontal and vertical ProS in PSP were slow, had irregular trajectories, and were prematurely interrupted; main sequence, acceleration, and deceleration were significantly below HC.
[52]	2022	PSP-RS (*n* = 18), PSP-C (*n* = 6), PSP-P (*n* = 6), PSP-PGF (*n* = 2), HC (*n* = 38)	EOG	68.6	NA	ProS, MGS	Severity of abnormalities: PSP-C > PSP-RS > PSP-P > PSP-PGF (nearly normal)
[22]	2008	PSP-RS (*n* = 12), PSP-P (*n* = 5), PD (*n* = 27), HC (*n* = 23)	VOG	64.1	2.7	ProS	Both PSP-RS and PSP-P had significantly slower ProS than PD and HC.
[83]	1994	PSP (*n* = 10), PD (*n* = 14), MSA (*n* = 14), CBD (*n* = 10), HC (*n* = 12)	EOG	62.5	2.1	GS, AS	GS and AS abnormalities can be used to differentiate PSP from PD, MSA and CBD.
[25]	2025	PSP-RS (*n* = 65), PSP-P (*n* = 39), PSP-PGF (*n* = 7), PSP-F (*n* = 1), PD (*n* = 50), HC (*n* = 50)	VOG	69.6	3.6	ProS	Vertical peak velocity is the best parameter for differentiating PSP from PD. Prolonged horizontal latency is associated with worse cognitive dysfunction of PSP.
[84]	2000	PSP (*n* = 10), CBD (*n* = 6), HC (*n* = 10)	EOG	69.1	2	GS, AS	Consecutive saccade recordings help diagnose PSP and atypical CBD earlier.
[85]	2022	PSP (*n* = 11), HC (*n* = 20)	VOG	46–76	4.5	GS, OS, MGS	In PSP, volitional saccades are slower and less accurate than reflexive saccades.
[86]	1989	PSP (*n* = 40), HC (*n* = 40)	EOG	68	2.8	GS, AS, OS	L-DOPA had no effect on latency in PSP.
[87]	2012	PD (*n* = 105), PSP (*n* = 11), MSA-P (*n* = 19), HC (*n* = 38)	VNG	77	2.7	ProS	Early ProS parameters cannot differentiate PSP, PD, MSA-P.
[88]	2023	PSP-RS (*n* = 16), PSP-P (*n* = 16), PSP-PGF (*n* = 3), PSP-F (*n* = 2)	VOG	72.6	4.5	ProS, AS	SC plays a key role in saccadic dysfunction in early PSP.
[89]	2019	Prob-PSP-RS (*n* = 5), PD-PIGD (*n* = 5), HC (*n* = 5)	camera-less computerized behavioral paradigm	70.2	3.7	ProS	Vertical/horizontal latencies correlated strongly with clinician-rated velocity in the corresponding direction.
[90]	2017	PSP-RS (*n* = 8), HC (*n* = 10)	VOG	74.3	4.2	ProS, AS	Vertical AS may be a useful parameter for detecting PSP.
[91]	2021	Prob-PSP (PSP-RS (*n* = 66), PSP-P (*n* = 28), PSP-PGF (*n* = 29), PD (*n* = 80)	VOG	68.0	3.7	ProS	The severity of oculomotor dysfunction can be used to differentiate clinical subtypes of PSP; it is significantly associated with dysphagia severity and SCP atrophy in PSP.

Prob-PSP, probable PSP; poss-PSP, possible PSP; so-PSP, suggestive PSP; VS, visual fixation suppression; SIFSs, small involuntary fixational saccades; L-DPOA, levodopa.

**Table 4:** Key diagnostic features of ProS for differentiating PSP from PD.

Candidate Biomarkers	Diagnostic Peformance
vertical peak velocity ↓	AUC 1.00; cut-off 111.7°/s; Se 100% (10/10); Sp 100% (11/11) [70]
vertical amplitude ↓	AUC 0.99; cut-off 1.68°; Se 100%; Sp 90.9% (10/11) [70]
downward amplitude ↓	cut-off 13.1°; Se 80.4%; Sp 93.8%; accuracy 89.6% [17]
mean vertical amplitude ↓	cut-off 12.6°; Se 92.2%; Sp 84.1%; accuracy 86.6% [17]
AxV	AUC 0.97; cut-off 3174°^2^/s; Se 84.3%; Sp 94.7%; accuracy 91.5% [17]
vertical latency ↑	cut-off 305–345 ms [89]
vertical duration	AUC 0.77; cut-off 21.6 ms; Se 70%; Sp 81.8%) [70]
(vertical mean velocity/horizontal mean velocity) × (vertical gain/horizontal gain)	AUC 0.96; cut-off 0.47; Se 76.9%; Sp 93.3% [79]
saccadic Go/No-Go	AUC 0.92; true-positive rate 0.73; precision 0.82 [110]

AUC, area under the curve; Se, sensitivity; Sp, specificity.

## Data Availability

No new data were created or analyzed in this study. Data sharing is not applicable to this article.
